# Barriers for Compliance to Breast, Colorectal, and Cervical Screening Cancer Tests among Hispanic Patients

**DOI:** 10.3390/ijerph13010021

**Published:** 2015-12-22

**Authors:** Christine Miranda-Diaz, Elba Betancourt, Yelitza Ruiz-Candelaria, Robert F. Hunter-Mellado

**Affiliations:** Universidad Central del Caribe, School of Medicine, Cancer Research Unit, Internal Medicine Department, P.O. BOX 60327, Bayamon 00960-6032, Puerto Rico; ismabet@yahoo.com (E.B.); yelitza.ruiz@uccaribe.edu (Y.R.-C.); robert.hunter@uccaribe.edu (R.F.H.-M.)

**Keywords:** cancer screening, compliance, barriers, Hispanics

## Abstract

Hispanics are less likely to undergo screening tests for colorectal cancer and cervical cancer than non-Hispanic whites. Compliance with mammography, fecal occult blood testing (FOBT), colonoscopy, and cervical smears (PAP) and barriers for compliance were studied. A descriptive study was performed with 194 ambulatory patients while they attended routine medical visits. Women are more likely than men to undergo a colonoscopy. Conversely, FOBT was most likely reported by men. Reasons for compliance with FOBT differed by gender. Men were most likely to avoid FOBT due to lack of knowledge whereas women reported that physicians do not recommend the procedure. Both men and women reported that lack of physician’s recommendation was their primary reason for not undergoing a colonoscopy. Men tend to report lack of knowledge about colonoscopy procedure. A higher mammogram utilization rate was reported by women older than 40 years. PAP smears were reported by 74% of women older than 21 years. The major reasons for avoiding mammography and PAP tests were having a busy schedule, fear, and feeling uncomfortable during the procedure. In a multivariate regression analysis, occupational status was found to be a predictor for compliance with FOBT and colonoscopy.

## 1. Introduction

Cancer is one of the leading causes of death worldwide. Over 1.6 million new cancer cases will be diagnosed and 589,430 people will die from the disease in the United States by the end of 2015 [1]. Early detection of cancer has been recommended to decrease the therapeutic burden associated with cancer therapy and to improve cancer prognosis and survival. Despite the availability and efficacy of cancer screening tests, patients are not undergoing preventive screening and often present with advanced cancer stages. For women at average risk for cervical and breast cancers an annual PAP smear beginning at age 21 and annual mammography beginning at age 40 are recommended [1]. Preventive colorectal cancer screening for men and women at average risk for colorectal cancer include an annual fecal occult blood testing (FOBT), sigmoidoscopy every 5 years, and colonoscopy every 10 years beginning at age 50 [1]. Data from the 2013 National Health Interview Survey (NHIS) revealed that the utilization rate of mammogram (72.6% *vs.* 81.1%), PAP test (80.7% *vs.* 93.0%), and colon cancer screening tests (58.2% *vs.* 70.5%) are below the 2020 Healthy People targeted utilization percentage [2]. Disparities in cancer diagnosis and screening test utilization by race have been reported among African American, Asian, and Hispanic populations mostly due to lack of health insurance and access to care [3,4,5,6,7].

Colorectal cancer screening test uptake was lower among Hispanic subgroups, except for Puerto Ricans in US mainland [8]. Lack of knowledge about colorectal cancer screening test, not recommended by Physicians, old age, male gender, high academic attainment, high income, and being White non-Hispanic among other factors were barriers for colorectal cancer screening [9,10]. Compliance with FOBT was not associated with race/ethnicity whereas FOBT is less frequently reported by women [7]. Lack of compliance with breast cancer and cervical cancer screening tests are more predominant among minorities. Hispanic women are less likely to receive a Physician’s recommendation for breast cancer, cervical cancer, and colorectal cancer screening interventions [11]. Other barriers for lack of compliance among Hispanic women are lack of health insurance, age, and usual source of care [6]. In a multivariate analysis, routine care visits in the last 12 months, Physicians’ recommendations, and having health insurance predicted compliance with breast and cervical cancer screening among Latinas [10,12]. Acculturation and place of birth have been related with compliance with colorectal cancer screening among minority population [12]. Demographic factors including marital status have been related with compliance with cervical cancer screening test in a group of Hispanic migrant farmworkers that have lived at least 5 years in US mainland [13].

Community-academia partnerships have been essential to improve compliance with cancer screening tests among Hispanic women [14]. Health education intervention should be developed to increase Physician’s recommendations for cancer screening test and to increase population awareness and knowledge about these tests. The purpose of our study was to evaluate the utilization rate and compliance with standard screening tests for breast, colon, and cervix cancers in a sample of Hispanic men and women in one of the metropolitan areas of Puerto Rico. We also assessed barriers for compliance with these screening tests.

## 2. Experimental Section

### 2.1. Subjects and Data Collection

This cross-sectional study included men and women between 21 and 85 years old. Eligible participants were approached while they attended or accompanied subjects to routine visits at two ambulatory clinics in Bayamon, PR. After informed consent was signed participants completed a brief questionnaire. The data collection was completed from April to September 2012. A total of 200 patients were recruited, 6 subjects were excluded due to a history of cancer more than two years ago. A total of 194 subjects completed the research questionnaire. The study was approved by the IRB from the Universidad Central del Caribe-School of Medicine.

### 2.2. Measures

A brief questionnaire was constructed to examine breast cancer screening, cervical cancer screening, and colorectal cancer screening practices among the subjects based on the literature review. The questionnaire included demographic, cancer history, and cancer screening behavior, and barriers for compliance with cancer screening tests variables: age, gender (*male/female*), weight, height, education *(less than high school education/high school education or higher)*, occupation *(employed/unemployed),* medical insurance *(government/Medicare/private/none),* annual income, number of routine visits per year, family history of cancer (*yes/no*), colorectal cancer diagnosis (*yes/no*), breast cancer diagnosis (*yes/no*), cervical cancer diagnosis (*yes/no*), previous diagnosis of intestinal polyps (*yes/no*), ulcerative colitis (*yes/no*), abnormal rectal bleeding (*yes/no*), breast mass (*yes/no*), compliance with mammogram (*yes/no*), FOBT (*yes/no*), colonoscopy (*yes/no*), and cervical tests (PAP) (*yes/no*). Barriers to comply with FOBT and colonoscopy in men and women older than 50 years, with mammogram in women older than 40 years, and with PAP in women older than 21 years included: *not recommended by physicians, didn’t know it had to be done, fear, not necessary, have no time, feel uncomfortable, don’t know what the procedure is, and health insurance don’t cover the procedure*. Barriers to comply FOBT and colonoscopy procedures in men and women less than 50 years old included: *family history of colorectal cancer, prior colorectal cancer diagnosis, prior ulcerative colitis diagnosis, and presence of abnormal rectal bleeding*. Barriers to comply with mammogram in women less than 40 years old included: *family history of breast cancer, breast mass, and breast pain*.

### 2.3. Data Analysis and Certification of Methods

Frequency and descriptive statistics were used in order to describe the data. Proportions (95% CI) of the barriers for screening practices were also estimated. Multivariate logistic regression analyses were conducted to predict lack of compliance with cancer screening practices using health insurance, family history of cancer, education, income, employment status, and gender as predictors. The significance level was set at *p* ≤ 0.05. SPSS 19 software (IBM Corp., Armonk, NY, USA) and Stata 12.1 software (StataCorp LP, College Station, TX, USA) were used to perform all the analyses. The DMSRSU research staff performed the statistical analyses.

## 3. Results and Discussion

Table 1 summarizes demographic variables and cancer screening practices. The majority of the sample were females (62.9%, *n* = 122). About 80% (*n* = 156) completed high school education or higher and more than half were unemployed (52.1%) with an annual income of less than $30,000 dollars (78.9%). The median age was 53.4 ± 15.0 years ranging from a minimum age of 21 to maximum age of 85 years. More than half of the sample had a private health insurance (54.1%). The median visits to a primary care physicians was 3.54 ± 4.24 whereas the median visits to a subspecialists was 2.77 ± 3.86 per year. Only 24% reported family history of cancer and 4% had a diagnosis of cancer no longer than 2 years ago. Twenty percent reported of the sample intestinal polyps. Among females, 21% reported a breast mass.

### 3.1. FOBT Test

Sixty percent of men and women older than 50 years (*n* = 114) have had a FOBT test in the last two years. FOBT test was reported by 67% of men and 57% of women. Table 2 shows gender differences for not undergoing a FOBT screening test. Women do not undergo a FOBT test mainly because it was not recommended by physicians (50%), have no time for the test (17%), and feeling uncomfortable with the test (7%). For men, reasons for not undergoing a FOBT test included: did not know it had to be done (53%) and lack of knowledge (33%). Sixteen percent of men and women less than 50 years old (*n* = 13) underwent a FOBT test due to the presence of abnormal rectal bleeding (61.5%, *n* = 8).

### 3.2. Colonoscopy

Fifty-four percent of men and women older than 50 years (*n* = 114) have had a colonoscopy in the last 10 years. Colonoscopy was reported by 42% of men and 62% of women. Table 2 shows gender differences for not undergoing a colonoscopy. For women, reasons for not undergoing colonoscopy test included: not recommended by physicians (54%), lack of time for the procedure (27%), feeling uncomfortable with the procedure (15%), and did not know it had to be done (15%). For men, reasons for not undergoing colonoscopy test included: not recommended by physicians (50%), lack of knowledge (31%), and did not know it had to be done (27%). Twenty-one percent of men and women less than 50 years old (*n* = 17) underwent colonoscopy procedure due to the presence of abnormal rectal bleeding (23%, *n* = 4), and family history of colon cancer (12%, *n* = 2).

### 3.3. Mammogram

Women older than 40 years (*n* = 102) 85% have had a mammogram in the last two years. Table 2 shows the most common reasons for not undergoing a mammogram: not having time (67%), fear (20%), and feeling uncomfortable with the procedure (13%). Thirty percent of women less than 40 years old (*n* = 6) have had a mammogram. The most common reason for undergoing a mammogram in female patients less 40 years old were presence of breast mass (15%) and family history of breast cancer (10%).

**Table 1:** Demographic factors and cancer screening practices.

Variables	M ± SD
Age	53.47 ± 15.00
Number of visits to primary care provider	3.54 ± 4.24
Number of visits to subspecialist	2.77 ± 3.86
Gender	
Male	37.1%
Female	62.9%
Education	
No high school diploma	19.6%
High school diploma or higher degree	80.4%
Employment	
Unemployed	52.1%
Employed	47.9%
Income	
<$29,000	78.9%
≥$30,000	21.1%
Health insurance	
Government/Medicare	41.2%
Private	54.1%
No insurance	4.6%
Family history of cancer	
Yes	23.7%
No	76.3%
Cancer diagnosis	
Yes	4.1%
No	95.9%
Mammography (women > 40 years) (*n* = 102)	
Yes	85.3%
No	14.7%
FOBT (>50 years) (*n* = 114)	
Yes	60.5%
No	39.5%
Variables	
Colonoscopy (>50 years) (*n* = 114)	
Yes	54.4%
No	45.6%
PAP (women > 21 years) (*n* = 122)	
Yes	73.8%
No	26.2%

### 3.4. PAP Test

Seventy-four percent of women (*n* = 122) have had a PAP test in the last two years. Table 2 shows the most common reasons for not undergoing a PAP test: not having time (56%), feeling uncomfortable (19%), and felt it was not necessary (9%).

**Table 2:** Barriers for not undergoing cancer screening tests (95% CI).

Barriers	Gender	
	Males	Females
**No FOBT (>50 year) (*n* = 45)**		
Physician not recommended	26.7 (2.8–50.5)	50.0 (31.3–68.7)
Didn’t know it had to be done	53.3 (26.5–80.2)	13.3 (0.6–26.1)
Fear	0	0
Not necessary	6.7 (−6.8–20.1)	6.7 (−2.7–16.0)
Have no time	0	16.7 (2.7–30.6)
Feel uncomfortable	0	6.7 (−2.7–16.0)
Don’t know what the procedure is	33.3 (7.9–58.7)	6.7 (−2.7–16.0)
**No Colonoscopy (>50 year) (*n* = 52)**		
Physician not recommended	50.0 (29.9–70.1)	53.8 (33.8–73.9)
Didn’t know it had to be done	26.9 (9.1–44.7)	15.4 (0.9–29.9)
Fear	0	11.5 (−1.3–24.4)
Not necessary	15.4 (0.9–29.9)	0
Have no time	7.7 (−3.0–18.4)	26.9 (9.1–44.7)
Feel uncomfortable	7.7 (−3.0–18.4)	15.4 (0.9–29.9)
Don’t know what the procedure is	30.8 (12.2–49.3)	3.8 (−3.9–11.6)
**No Mammography (>40 year) (*n* = 15)**		
Physician not recommended		6.7 (−7.6–21.0)
Didn’t know it had to be done		0
Fear		20.0 (−2.9–42.9)
Not necessary		6.7 (−7.6–21.0)
Have no time		66.7 (39.6–93.7)
Feel uncomfortable		13.3 (−6.2–32.8)
Don’t know what the procedure is		6.7 (−7.6–21.0)
**No PAP (>21 year) (*n* = 32)**		
Physician not recommended		6.3 (−2.6–15.1)
Didn’t know it had to be done		3.1 (−3.2–9.5)
Fear		6.3 (−2.6–15.1)
Not necessary		9.4 (−1.3–20.1)
Have no time		56.3 (38.1–74.4)
Feel uncomfortable		18.8 (4.5–33.0)
Don’t know what the procedure is		3.1 (−3.2–9.5)

Table 3 shows that being employed is a predictor of lack of compliance with FOBT test (χ^2^ = 18.66, *p* < 0.000 with *df* = 1). Prediction success overall was 66.5%. Employed men and women older than 50 years in the sample were 0.17 times more likely to avoid a FOBT test. Being employed and having a higher income were found as predictor of lack of compliance with colonoscopy (χ^2^ = 15.35, *p* < 0.000 with *df* = 1; χ^2^ = 5.43, *p* < 0.02 with *df* = 1, respectively). For mammogram and PAP test no significant predictor for lack of compliance with breast cancer and cervical cancer were found.

**Table 3:** Multiple logistic regression model for colorectal cancer screening test.

Predictors	*p*	OR	95% CI	
			LL	UL
**FOBT**				
Employed	0.00	0.17	0.07	0.38
Constant	0.45	1.36		
**Colonoscopy**				
Employed	0.00	0.19	0.08	0.44
≥$30,000	0.02	2.93	1.19	7.22
Constant	0.15	0.55		

## 4. Discussion

Cancer screening practices among the Hispanic population have been reported elsewhere. The utilization rate for breast cancer, cervical cancer, and colorectal cancer in our sample is below the 2020 Healthy People targeted utilization percentage. In our sample, women were more likely than men to undergo a colonoscopy. Conversely, FOBT was frequently reported by men. Reasons for complying with FOBT and colonoscopy differed by gender. Men were most likely to avoid FOBT due to lack of knowledge whereas women reported that physicians do not recommend the procedure. Both men and women reported that lack of physician’s recommendation was their primary reason for not undergoing a colonoscopy. However, men tend to report lack of knowledge about colonoscopy procedure. In a multivariate regression analysis, occupational status was found as a predictor for compliance with FOBT and colonoscopy. Our data is concordant with those reported by and colleagues.

Different from the Wells and Roetzheim data [8], our cohort had higher mammogram (85%) utilization rates in in females older than 40 years. The major reasons for avoiding mammography and PAP tests were having a busy schedule, fear, and feeling uncomfortable during the procedure. Conversely, in other Hispanic populations, not having a physician recommendation for cancer screening tests was the primary reason for not doing a mammogram and PAP test [5]. For Latinas living in California, lacking health insurance, being older, and usual source of care were the primary reasons for a delaying a mammogram [9].

## 5. Conclusions

In our cohort of Hispanic Puerto Rican subjects, occupational status was found to be a predictor for lack of compliance with colonoscopy and FOBT. Tailored health education interventions directed to describe the nature and benefit of cancer screening tests might improve compliance as well as educating health care providers about the importance of referral. Health campaigns presented in the work environment that are directed at increasing cancer screening would also enhance the compliance with these studies. Our study is limited by the small sample size and may not be generalizable to the entire population of the island.
